# Generation and characterization of a superior host cell line for biomanufacturing

**DOI:** 10.1186/1753-6561-9-S9-P13

**Published:** 2015-12-14

**Authors:** Anett Ritter, Johannes Wienberg, Bernd Voedisch, Burkhard Wilms, Sabine Geisse, Thomas Jostock, Holger Laux

**Affiliations:** 1 Novartis Institutes for BioMedical Research, Basel, Switzerland; 2 Novartis Pharma AG, Basel, Switzerland; 3 Chrombios, Nussdorf, Germany

## Background

Chinese hamster ovary (CHO) cells are the most widely used host for large scale production of recombinant therapeutic proteins exhibiting high productivities in the gram per liter range. Although these cells have been extensively characterized and optimized, a demand to further increase the performance towards higher productivity and clonal stability exists. Up to date, the clone selection process is mainly based on phenotypic screens like titer measurements and growth data instead of more reliable biomarkers. Recently, the genomes of Chinese hamster as well as of different CHO cell lines were sequenced, assembled and annotated [1-3]. This new information gives the opportunity for functional analysis of the transcriptome and allows rational designs for the identification of biomarkers for clone selection and targets for cell line engineering.

## Material and methods

CHO-K1a and CHO-C8DEL cell lines were cultivated in shake flasks in a non-humidified shaker cabinet at 150 rpm, 10% CO2 at 36.5°C in suspension in proprietary, chemically defined culture media. Cell viabilities and growth rates were monitored by means of an automated system (ViCell, Beckman Coulter).

For transient expression plasmid DNA and polyethylenimine "Max" (Polysciences, Eppelheim, Germany) were diluted into OptiMEM medium (Life Technologies) and added to the cells after complex formation. The titer of the model protein in the supernatant was measured by Protein A HPLC on day 3 and day 6 post transfection.

Cells were stably transfected by electroporation (Amaxa Nucleofection system, Lonza, Germany) with an expression plasmid encoding for a human monoclonal antibody according to the manufacturer's instructions.48 h after transfection, selection was started by addition of geneticin (G418). As soon as cells recovered to a viability of above 80%, a second selection step was applied by passaging the cells into G418-free medium containing 1 µM methotrexate (MTX). When the cells resumed logarithmic growth, cell cultivation was continued in MTX-containing medium throughout single cell sorting, screening and productivity assessments. Cells were stained using a FITC-labeled mouse IgG-anti-human IgG (BD Pharmingen) and single cell sorting was performed using a FACS-Aria II instrument (Becton Dickinson). The 5% most strongly fluorescent cells were gated and sorted as single cells into 96-well plates. Volumetric productivities of G418 and MTX selected pools and clones were determined by Protein A HPLC in cell culture supernatants.

## Results

Analysis of gene expression profiles of 5-7 high and 5-7 low monoclonal antibody producing CHO-K1a clones of three different monoclonal antibody projects surprisingly revealed that the telomeric region of chromosome 8 including several genes is often lost in transfected, high antibody producing CHO-K1a derived clones. Likewise surprisingly, a high correlation between the loss of this telomeric region and increased productivity as well as clonal stability could be identified in CHO-K1a clones.

**Figure 1:**
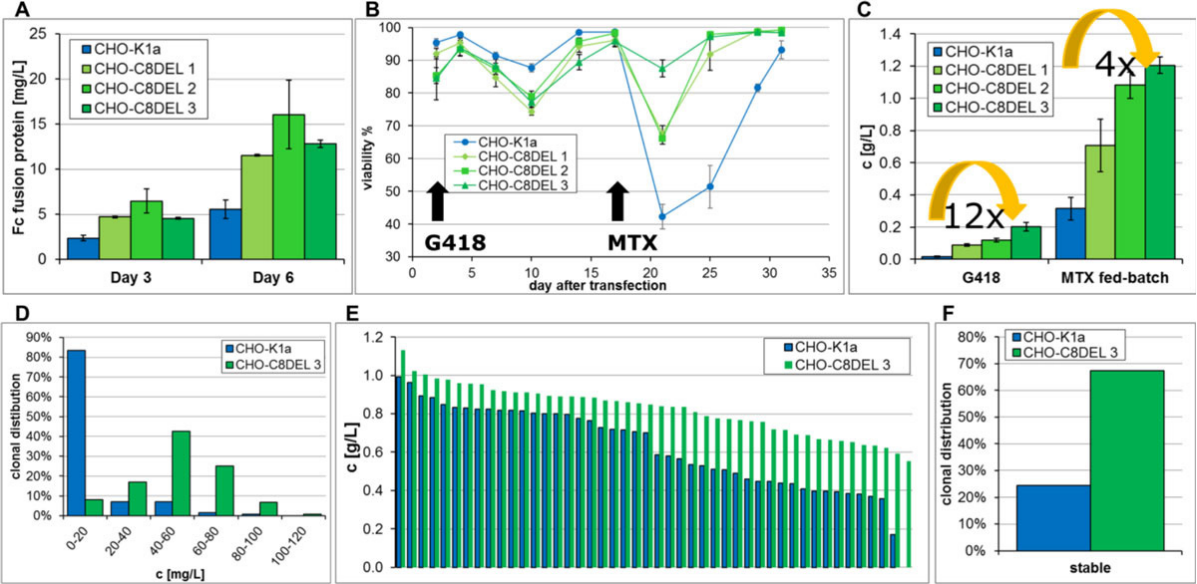
**(a): Comparison of productivities of an Fc-fusion protein after transient transfection**. Average of 3 pools ± STDev.(b): Viabilities after transfection (day 0) and during selection for three CHO-C8DEL cell lines (green) compared to the CHO-K1a cell line (blue) expressing a monoclonal antibody. Average of 4 pools ± STDev. (c): After both selection steps a massive increase of productivity of a monoclonal antibody could be detected for all three CHO-C8DEL lines (green) compared to the CHO-K1a cell line (blue). Average of 4 pools ± STDev.(d): After single cell cloning of high producing pools of CHO-C8DEL 3 and CHO-K1a, significantly more high producing cells were isolated for the CHO-C8DEL 3 cell line. E: Productivity of 45 best producing CHO-K1a and CHO-C8DEL 3 clones. F: Comparison of stability of 45 best producing CHO-C8DEL 3 clones compared to 37 best producing CHO-K1a clones over a cultivation period of 12 weeks.

The aim of this study was to develop a new parental cell line lacking this telomeric region. Three of such clones could be isolated and were named CHO-C8DEL 1-3. The capability of these isolated, new parental cell lines for cell line development and protein production was subsequently evaluated in depth: Volumetric pool productivities are significantly increased after transient (Figure 1A) and stable transfections (Figure 1C) in the three newly generated CHO-C8DEL parental cell lines. Additionally, the drop of cell viability during MTX selection is less pronounced and cells recovered 7-8 days faster from crisis (Figure 1B). Moreover, significantly more high and stable producing clones were retrieved after single cell sorting (Figure 1D), facilitating the screening efforts in order to obtain a comparable number of high and stable production clones. Also, the volumetric productivity(Figure 1E) as well as stable production over a period of 12 weeks of the CHO-C8DEL clones is increased (Figure 1F, clones were classified as stable if they were losing not more than 25% of productivity).

## Conclusions

With our study we contribute to the knowledge of how the recently published CHO genomes [1-3] can be used to meet the growing demand of industry to further develop CHO cell lines and to increase their performance for the production of new therapeutic proteins in short time periods.

We compared gene expression profiles of high and low antibody producing CHO-K1a clones and identified a subset of genes, localized at the telomeric region of chromosome 8, which have a role regarding productivity and production stability. These genes can be used as biomarkers for selecting high and stable producing cell lines. In addition, we generated and characterized a new parental CHO cell line lacking this telomeric region, which displays several advantageous characteristics for cell line development and manufacturing.
